# Climate and landscape mediate patterns of low lentil productivity in Nepal

**DOI:** 10.1371/journal.pone.0231377

**Published:** 2020-04-16

**Authors:** Gokul P. Paudel, Mina Devkota, Alwin Keil, Andrew J. McDonald

**Affiliations:** 1 International Maize and Wheat Improvement Center (CIMMYT), South Asia Regional Office, Kathmandu, Nepal; 2 International Maize and Wheat Improvement Center (CIMMYT), National Agricultural Science Centre Complex (NASC), New Delhi, India; Institut de Recherche pour le Developpement (IRD), FRANCE

## Abstract

Lentil (*Lens culinaris* Medik.) is a cool-season pulse grown in winter cropping cycle in South Asia and provides a major source of nutrition for many low-income households. Lentil productivity is perceived to be sensitive to high rainfall, but few studies document spatial and temporal patterns of yield variation across climate, soil, and agronomic gradients. Using farm survey data from Nepal, this study characterizes patterns of lentil productivity and efficiency for two cropping seasons. Additional insights were derived from on-farm trials conducted over a 5-year period that assess agronomic, drainage, and cultivar interventions. To contextualize the inferences derived from farm surveys and trials, the Stempedia model was used to simulate the severity of *Stemphylium* blight (*Stemphylium botryosum*) risk–the principal fungal disease in lentil–with 30 years of historical climate data. Although development efforts in Nepal have prioritized pulse intensification, results confirm that lentil remains a risky enterprise highlighting the prevalence of crop failures (16%), modest yields (353 kg ha^-1^), and low levels of profitability (US$ 33 ha^-1^) in wet winters. Nevertheless, site factors such as drainage class influence responses with upland sites performing well in wet winters and lowland sites performing well in dry winters. In wet winters, a phenomena perceived to be increasing, 76% of surveyed farmers reported significant disease pressure and simulations with Stempedia predict that conditions favoring *Stemphylium* occur in >60% of all years. Nevertheless, simulation results also suggest that these risks can be addressed through earlier planting. Based on the combined results, gains in yield, yield stability, and technical efficiency can be enhanced in western Nepal by: 1) ensuring timely lentil planting to mitigate climate-mediated disease risk, 2) evaluating new lentil lines that may provide enhanced resistance to diseases and waterlogging, and 3) encouraging the emergence of mechanization solutions to overcome labor bottlenecks.

## Introduction

In South Asia, climate change impacts are increasingly apparent, with drought [1,2], floods and waterlogging [3–7], and heat waves [8,9] all increasing. Plethora of literature have shown that these climatic anomalies in South Asia will continue in the future [10–20]. With more climatic anomalies and progressive climate changes, many studies have documented either an increased occurrence frequency or new degree of severity of abiotic stresses affecting crop growth and yield [21–28]. Climate changes are also influencing the spread and intensity of biotic stresses including diseases, weeds, and pests [25,26,29,30]. Moreover, there can be interactions between biotic and abiotic stresses that increase crop damage [21,22,31].

Lentil (*Lens culinaris* Medik.) is one of the major winter pulse crops in South Asia and is typically sown after rice in the annual cropping rotation. Lentil cultivation in South Asia constitutes almost half of the area and one third of the volume of global production [32]. Lentil seed contains high concentrations of protein and micronutrients [33], thereby playing a major role in the food and nutritional security of millions of low-income South Asian families [34], and is also an important contributor to soil health in cereal-based cropping systems due to its nitrogen fixing ability [35,36]. In Nepal, lentil is grown in the Terai, Inner Terai and mid-hills and constitutes 60% of the total grain legume area and production [37]. Globally, Nepal ranks sixth in terms of lentil production and fifth in terms of export to world markets [32,37]. Global demand for lentil has expanded at a robust annual rate of 6.2% over the past ten years, and it is estimated that export revenue derived from lentil in Nepal could double or even triple if relevant actions are taken to boost cultivated area, productivity, and market integration [38,39].

However, in recent years lentil yields in Nepal have stagnated due to factors including low levels of investment in inputs, cultivation of older varieties, and increased disease pressure [40–45]. In Nepal, lentil is cultivated under rainfed conditions after the monsoon rains have retreated, and production is highly sensitive to growing season weather [35,36,46,47]. Lentil productivity, therefore, is dependent on the residual soil moisture available from the late monsoon season and the amount and distribution of winter rainfall. However, excessive seasonal rainfall increases the chances of waterlogging in lentil particularly in lowland areas with poor drainage facility. In higher rainfall winters, waterlogging both directly impedes lentil growth and development while potentially causing or exacerbating fungal disease outbreaks [36,48]. Erskine and El Ashkar [47] reported that variability in rainfall during the lentil production period is responsible for 41% of the variation in mean yields. Furthermore, in recent years, the fungal disease *Stemphylium* blight (*Stemphylium botryosum*), has emerged as a major threat to lentil production in South Asia, including in Nepal [49,50]. Consequently, lentil yields in Nepal are judged to be highly sensitive to both rainfall deficit and rainfall excess, extremes that some projections suggest may increase in the future [51]. High sensitivity to variable climate risks is also perceived to be a major barrier towards farmer investments in productivity enhancement.

Minimizing lentil yield losses has emerged as a development priority for improving livelihoods and securing the food and nutritional security in Nepal. In order to meet the demand for lentil, either production or productivity have to be increased. Expanding the land frontier to increase production is not considered a viable option due to low availability of land reserves that could be brought into production with acceptable environmental costs [52,53]. Hence, the most viable option for enhancing lentil production is through improving productivity levels.

For the study region in the Mid- and Far-western Terai plain and mid-hill region of Nepal (henceforth ‘western’ Nepal), this study integrates three complementary research activities: 1) household surveys (2014 and 2015 harvests) to assess current productivity levels and drivers of technical efficiencies, 2) on-farm trials to assess the value of new agronomic production practices and varieties (2012/13–16/17), and 3) simulations with the Stempedia model [49] to assess inter-annual variations in *Stemphylium* disease risk and to establish the risk-reducing value of modifying planting dates.

## Materials and methods

This research uses different types of dataset and followed the standard ethics during the data collection. First, no personal identifiable information was recorded in the experimental trails data. Only farmers’ plots were used to establish experimental trials in the study areas, after taking consent from the farmers to use their plots. Second, for the farm survey, the authors followed the donor’s standard ethics protocol regarding the human subject research during the data collection. The authors have also taken the consent from the farmers before the survey so that no any personal identifiable information would be disclosed during data sharing.

### Site description

On-farm experiments were conducted over five seasons (2012–2016 harvest years) to evaluate the influence of agronomic interventions and varietal choice on lentil yields in two contrasting ecologies: the mid-hills (Surkhet and Dadeldhura districts) and the Terai plain (Banke, Bardiya, Kailali, and Kanchanpur districts). The two districts in the mid-hills are located at 28.01° to 28.59° E latitude and 80.12° to 81.36° N longitude and 700–1800 m altitude, while the four districts in the lowland Terai are located at 27.51° to 29.28° E latitude and 80.01° to 82.8° longitude and range of 100–700 m altitude. The experimental sites in the mid-hills are well-drained (upland) with maize (April-August) and lentil (September to April) grown in the annual rotation, while the Terai sites are poorly drained (lowland) with rice (June-October) and lentil (October-March) grown in the annual cropping system rotation.

### On-farm experiments

Three categories of on-farm experiments were conducted: (1) evaluation of different crop establishment methods: drainage modification (bed planting), zero tillage, minimum tillage (single pass), and hand broadcasting into conventionally-tilled soil, (2) varietal evaluations using recently released varieties by Government of Nepal, advanced genotypes (in the process of release) and locally available varieties grown by the farmers, and (3) integrated management, i.e., improved variety, fertilizer application (recommended rates of 20:40:20 kg N:P_2_O_5_:K_2_O ha^-1^), mechanized seeding contrasted to prevailing farmer practices of hand broadcasting of local seed with conventionally-tilled soil and no mineral fertilizer. Details of the year and location for these experiments are presented in S1 Table. In each of the districts experimental areas were located at 1 to 25 km distance. For each experiment, analysis of variance (ANOVA) was conducted to assess treatment and year effects along with their interaction.

### Household surveys

The survey was conducted for two lentil producing years (2013–14 and 2014–15, hereafter referred as 2014 and 2015, respectively). In 2014, the survey was conducted in four major lentil growing districts: Banke and Bardyia in the Mid-western Terai region along with Kailali and Kanchanpur in the Far-western Terai region. Based on the lentil acreage in each of the sub-districts (village development committees or VDCs), 10 VDCs were purposively selected to capture major lentil growing areas. With these VDCs, a total of 193 lentil-growing households were randomly selected for the survey. In 2015, the survey was expanded to six districts by including two additional districts from the mid-hills, i.e., Surkhet (Mid-western region) and Dadeldhura (Far-western region). A total of 43 VDCs and 600 households were surveyed in 2015 using the same selection methodology as in the previous year.

Data were collected through face-to-face interviews using a structured questionnaire, conducted following lentil harvest. The questionnaire includes sections to elicit information on household demographics, lentil production technologies, inputs, agronomic practices, yields, end uses for harvested lentil, and general perceptions towards lentil cultivation. Households that planted lentil but lost the crop due to biotic or abiotic stress were retained in our analysis.

### Weather data

Daily minimum and maximum temperature (°C), and rainfall (mm) data corresponding to the household survey periods were collected from the Department of Hydrology and Meteorology, Government of Nepal [54]. Rainfall (mm) along with maximum and minimum temperatures (°C) were averaged for each weather station (*n = 16* for temperature, *n = 58* for precipitation) within our study area over the lentil season (October to March). An inverse distance weighting (IDW) algorithm [55] in ArcGIS v10.3 was used to create gridded weather data at a 1 km resolution. Gridded data were then paired with survey locations. Furthermore, long-term (1985–2015) historic weather data for selected meteorological stations from different districts of the Nepal Terai were also obtained from the same sources [54], and a historical trend analysis has been carried out for seasonal precipitation. Station data from Banke district was used to drive the Stempedia model simulations.

### Stochastic frontier model

A production frontier represents the maximum output attainable for a given set of inputs and a given production technology [56]. Failure to attain the frontier output implies that production is technically inefficient. However, survey data on agricultural production may be heavily contaminated by statistical noise due to measurement errors, variability in climatic and edaphic conditions, or interactions with pests and diseases. In contrast to Data Envelopment Analysis (DEA) that attributes any deviation from the frontier output to inefficiency [57], stochastic frontier analysis accommodates statistical noise. We therefore chose this approach for our analysis. The stochastic production frontier was independently proposed by Aigner et al., [58] and Meeusen and van Den Broeck [59] and is defined as follows:

$$Y_{i}=F(X_{i};\beta )\text{exp}\left(V_{i}-U_{i}\right),i=1,2,\ldots ,N$$ (1)

Where;

Y = Quantity (or value) of output of the i-th firm

F(·) = Suitable production function

X = Vector of input quantities

*β* = Vector of parameters to be estimated

*V* = Random error term

*U* = Non-negative error term representing technical inefficiency

V is a random variable, assumed to be independently and identically distributed as *N*(0, *σ*_*v*_). *U*, which captures systematic shortfalls from the frontier due to technical inefficiency, is assumed to follow a particular one-sided distribution. A number of different distributions have been proposed in the literature, namely the half-normal and exponential [58], the truncated normal [60], and the two-parameter Gamma distribution [61]. The technical efficiency (TE) measure for the i-th household *TE* = exp(−*U*_*i*_) ∈ [0, 1] is the ratio of the observed output and the maximum attainable output at the frontier. The maximum likelihood (ML) estimation of Eq (1) yields estimates of *β* and *γ* where,$\gamma =\frac{\sigma_{U}^{2}}{\sigma^{2}}\in [0,1]$, and $\sigma^{2}=\sigma_{U}^{2}+\sigma_{V}^{2}$. Hence, the model separates the residuals into a normally distributed random error and a one-sided error term reflecting technical inefficiency; the latter is related to input management and measures the degree to which a farmer was able to obtain the maximum possible output for a given vector of inputs.

Several studies have been previously conducted using the two-stage stochastic production frontier model. In the two-stage stochastic production frontier analysis, the first stage involves the specification and estimation of stochastic frontier and prediction of technical efficiency score, while in the second stage determinants of the technical efficiency score are regressed. However, Battese and Coelli [62] and Wang and Schmidt [63] reported that the two-stage method is inconsistent and a single equation method is preferred because the two-stage method contradicts the assumption of an independently, identically and normally distributed inefficiency effect in the stochastic frontier function. Furthermore, Wang and Schmidt [63] pointed out that the first stage is biased if dependent variables of the first and determinants of the second stage are correlated. Kumbhakar et al. [64] and Reifschneider and Stevenson [65] specified a stochastic frontier model in which inefficiency effects were defined to be explicit functions of firm-specific factors, and all parameters are estimated in a single-stage maximum likelihood procedure. In this study we applied the Battese and Coelli [62] model for the two years of cross-sectional data to derive unbiased estimates for lentil production in Nepal. Since both years were similar in terms of climatic parameters (wet winter), we amalgamated two years of survey data in order to develop a single production frontier. According to Battese and Coelli [62], the technical efficiency effects of U are obtained by truncation (at zero) of the normal distribution with mean μ_i_ = Z_i_δ, where Z_i_ is the farm specific explanatory variables and δ is the vector of parameters to be estimated.

The stochastic frontier production function for lentil farming has a Cobb-Douglas type functional form and the production function estimation adopted in our study is defined as:

$$\text{Ln}\;Y_{i}=\beta_{0}+\sum_{j=1}^{9}\beta_{j}Ln(X_{ij})+\sum_{k=1}^{9}\beta_{k}(D_{ik})+\sum_{p=1}^{1}\sum_{r=1}^{1}\beta_{pr}R_{pri}+V_{i}-U_{i}$$ (2)

Where, Ln Y_i_ = Natural logarithm of output

i = i-th farm (i = 1, 2, 3,…, 665)

β = vector of parameters to be estimated

Ln(X_j_) = vector of input parameters

D_k_ = vector of dummy variable related with lentil production

R_pr_ = Total amount of rainfall differentiated by lowland production ecology

V = N(0, σV distributed random error term

U = non negative error term that represents technical inefficiency

The non-negative error term is specified as a function of household-specific determinants of technical inefficiency, which can be specified as:

$$U_{i}=\delta_{0}+\sum_{l=1}^{7}\delta_{l}Z_{li}+W_{i}$$ (3)

Where, δ = vector of parameters to be estimated

Z_l_ = vectors of potential inefficiency determinants

W = N(0, *σ*_U_) distributed random error, where σ_U_ is defined such that U_i_ ≥ 0.

The STATA statistical program was used to estimate the maximum likelihood estimation of parameters for Eqs (2) and (3) based on a Cobb-Douglas type functional form.

Our dataset contained 128 cases of complete crop failure and, hence, reported zero yield; these cases were omitted from the stochastic frontier analysis in order to avoid biased estimates of the production function. However, to crosscheck the robustness of our findings we estimated a Tobit model [66], which can accommodate zero output (yield) data, in addition to the stochastic frontier production function. The Tobit model has been widely applied when the dependent variable contains an accumulation of observations at zero [67–71].

The definitions and summary statistics of all variables included in the model are presented in the Table 1. The quantity of the lentil grain yield harvested by the farmers is the dependent variable, which is regressed with a number of farm input variables. The farm input variables used in the current stochastic frontier analysis are of two types. The first type of variable included in the models are continuous in nature and are: cultivated lentil land, labor, non-labor capital, amount of seed, amount of nitrogen, phosphorus and potassium, and amount of rainfall and average temperature during the lentil production period. The second type of variables included in the models are dummy in nature and are related to soil types, diseases, waterlogging, variety, and production ecologies for lentil farming. Finally, we also included the interaction of rainfall with lowland production ecology in the model.

**Table 1:** Lentil production characteristics and summary statistics.

Variables	Variables description	Farms without crop failure (N = 665)		Full samples (N = 793)	
		Mean	Std. Dev	Mean	Std. Dev
Output^†^	Total lentil grains produced (kg)	89.72	165.55	75.24	155.14
Cultivated land^†^	Total cultivated land (ha)	0.70	0.72		
Land^†^	Area under lentil production (ha)	0.26	0.31	0.27	0.32
Capital inputs^†^	Total non-labor capital inputs (NRs) excluding seed and fertilizers	1489.32	2930.78	1568.49	2949.26
Household size	Number of household members (No.)	7.16	4.01		
Gender	Dummy, = 1 if household decision maker is male otherwise 0	0.80			
Farming experience (years)	Years of lentil farming (years)	11.95	12.6		
Labor^†^	Total hired and family labor used for lentil production (labor-hours)	101.79	118.54	104.35	117.64
Labor availability	Dummy, = 1 if labor is easily available	0.65			
Wage rate^†^	On farm wage rate; NRs/day (sub-district level indicator)	306.47	39.21		
Lentil specialized farms	Dummy, = 1 if farms cultivate lentil on ≥ 50% of their cultivated land	0.38			
*Input variables*					
Seed^†^	Seed quantity (kg)	11.38	13.72	11.36	13.35
Nitrogen^†^ (N*)	Total amount of Nitrogen applied (kg)	0.84	3.27	1.42	4.26
Phosphorus^†^ (P_2_O_5_*)	Total amount of Phosphorus applied (kg)	1.64	5.6	2.23	6.03
Potassium^†^ (K_2_O*)	Total amount of Potash applied (kg)	0.17	1.68	0.22	1.72
Variety	Dummy, = 1 if farms used improved varieties for lentil production otherwise 0	0.08		0.09	
Rainfall^†^	Total amount of rainfall during lentil production period October to March (mm), sub-district level	252.89	116.77	253.38	108.76
Waterlogging	Dummy, = 1 if lentil growing fields are waterlogged after rainfall otherwise 0	0.27		0.29	
Temperature^†^	Average temperature during lentil production (October to March) period (°C), sub-district level	18.51	1.86	18.6	1.73
Relay seeding	Dummy, = 1 if lentil is relay cropped with rice otherwise 0	0.07		0.09	
Mixed cropping	Dummy, = 1 if lentil is mixed cropped with other crops otherwise 0	0.55		0.57	
Tillage method	Dummy, = 1 if lentil growing plots are tilled using tractors and/or power tillers otherwise 0	0.35		0.36	
Diseases	Dummy, = 1 if lentil crop suffered from diseases and/or pest infestation otherwise 0	0.78		0.76	
Soil type (sand)	Dummy, = 1 if soil is coarse texture otherwise 0	0.20		0.19	
Soil type (silt)	Dummy, = 1 if soil is medium texture otherwise 0	0.42		0.43	
Soil (clay)	Dummy, = 1 if soil is fine texture otherwise 0	0.37		0.39	
Land type (lowland)	Dummy, = 1 if production ecologies lies in lowland otherwise 0 if it is upland	0.60		0.65	
Rainfall × lowland	Total amount of rainfall (mm) if the production ecology is lowland	131.66	132.39	146.36	131.91

^†^Variables used in logged form in the production efficiency and technical efficiency analysis.

*Urea, DAP and Potash are the fertilizers for the N, P_2_O_5_, and K_2_O used in the model. Exchange rate: 1 US $ = NRs 98.6 and NRs. 102.7, respectively, for year 2014 and 2015 [86].

The variables used for technical efficiency analysis are presented in Table 1 and most of the variables are included in the model as suggested by Bravo-Ureta and Pinheiro [72]. Some of the variables used in technical inefficiency determinant analysis are based on site-specific considerations. Inclusion of a gender variable in the model differentiates the potential role of gender in technical efficiency; in our case almost 80% of households were headed by a male. Furthermore, farming experiences influence the technical efficiency of lentil farming and we expect that farming experience is positively associated with technical efficiency. We included cultivated land and lentil-specialized farms (i.e. >50% of winter cropped area in lentil) as potential technical efficiency determinants. While we expect that cultivated land has an inverse relationship with technical efficiency as farmers with limited land resources may have incentives to increase yields through meticulous crop and input management [73], the lentil-specialized farms are expected to have a positive relationship. Finally, we included the on-farm labor wage rate and labor availability variables; the former is expected to have a negative relationship with technical efficiency while the latter is expected to have a positive relationship.

### Simulating *Stemphylium* blight disease severity simulation using the Stempedia model

The Stempedia model predicts the potential severity of *Stemphylium* blight disease and associated yield reductions in lentil based on sowing date, first flowering date, and daily weather; model performance has been verified in western Nepal [49]. The model assumes that inoculum does not limit disease progression. In our study, Stempedia is used to predict mean and inter-annual variations in disease severity risk in Banke district as a representative production environment for lentil in western Nepal. Crop characterization data was derived from on-farm and on-station experimental trials conducted in the region. To drive the model, long-term (1985–2015) weather data for rainfall and minimum and maximum temperature were used from Banke district, with additional data for solar radiation and relative humidity retrieved from NASA’s POWER datasets [74]. Lentil variety Khajura-2, commonly grown by farmers in the western Terai, was used for the simulations. The model was run for nine different seeding dates at 10-day intervals starting from 1^st^ October to 20^th^ December. Seeding time of lentil range from 3^rd^ week of October to end of November, hence the dates bracketed the plausible seeding window for lentil in the rice-based production systems of the Terai.

## Results

### Characterization of lentil production across environmental gradients in western Nepal

Average rainfall during the lentil growing season (October to March) was 253 mm and daily average temperature was 18.5°C (Table 1). Seasonal rainfall in the studied year was significantly higher than the long-term average and is reflected in the survey data with 27% of farmers reporting that their lentil fields were affected by waterlogging. The average land area under lentil production was 0.26 ha with a grain yield of 90 kg ha^-1^. In the region, 57% of farmers cultivated lentil in a mixed cropping system with mustard, wheat and *lathyrus*, and 7% of farmers practiced relay seeding of lentil with rice. The majority of farmers (91%) were using a local variety of lentil, with very low use of purchased fertilizers with average application rate for nitrogen, phosphorus, and potassium of 3.23, 6.31, and 0.65 kg ha^-1^, respectively. Sixty-five percent of farmers grew lentil in lowland areas which are dominated by clay to silty loam soils and vulnerable to waterlogging during periods of high rainfall. About 76% of farmers reported that their lentil field suffered from diseases (Table 1).

In relatively wet winters included in the survey, lentil productivity in upland ecologies with better drainage (397 kg ha^-1^) was significantly higher than in lowland ecologies (329 kg ha^-1^) (Table 2). On the other hand, although still modest, investment in fertilizer was significantly higher in lowland ecologies (US$ 12.8 ha^-1^) than in upland (US$ 2.4 ha^-1^). The total labor cost involved in lentil cultivation was significantly higher in the upland ecologies (US$ 201 ha^-1^) than in lowland (US$ 117 ha^-1^). The higher labor cost in upland ecologies is associated with limited mechanization options. However, the non-labor capital cost was significantly higher in lowland (US$ 57.8 ha^-1^) than in upland ecologies (US$ 46.6 ha^-1^). The total variable cost (US$ 295 ha^-1^ in upland vs. US$ 237 ha^-1^ in lowland) and gross margin (US$ 53.5 ha^-1^ vs US$ 22.2 ha^-1^) were significantly higher in the upland than in lowland ecologies. Despite having significantly higher yields and gross margins, the benefit–cost ratio is significantly lower in upland environments due to higher production costs.

**Table 2:** Lentil production characteristics differentiated by production ecologies.

Variables	Upland (N = 278)	Lowland (N = 515)	Sig.	Overall (N = 793)
Lentil yield (kg ha^-1^)	397.3	329.2	***	353.1
Seed cost ($ ha^-1^)	44.5	49.8	ns	47.9
Fertilizer cost ($ ha^-1^)	2.4	12.8	***	9.1
Labor cost ($ ha^-1^)	201.3	116.8	***	146.5
Non-labor capital ($ ha^-1^)	46.6	57.8	**	53.9
Total variable cost ($ ha^-1^)	294.7	237.3	***	257.4
Gross Revenue ($ ha^-1^)	348.2	259.5	***	290.6
Gross Margin ($ ha^-1^)	53.5	22.2	***	33.2
Benefit cost (B:C) ratio^†^	1.30	1.35	***	1.33

*** indicates significant at 1% level,

** indicates significant at 5% level, and “ns” indicates non-significant. Across production ecologies comparison are based on Mann-Whitney test. Exchange rate: 1 US $ = NRs 98.6 and NRs. 102.7, respectively, for year 2014 and 2015 [86].

^†^The benefit cost ratio was calculated by dividing gross revenue with the total variable cost.

**Fig 1:**
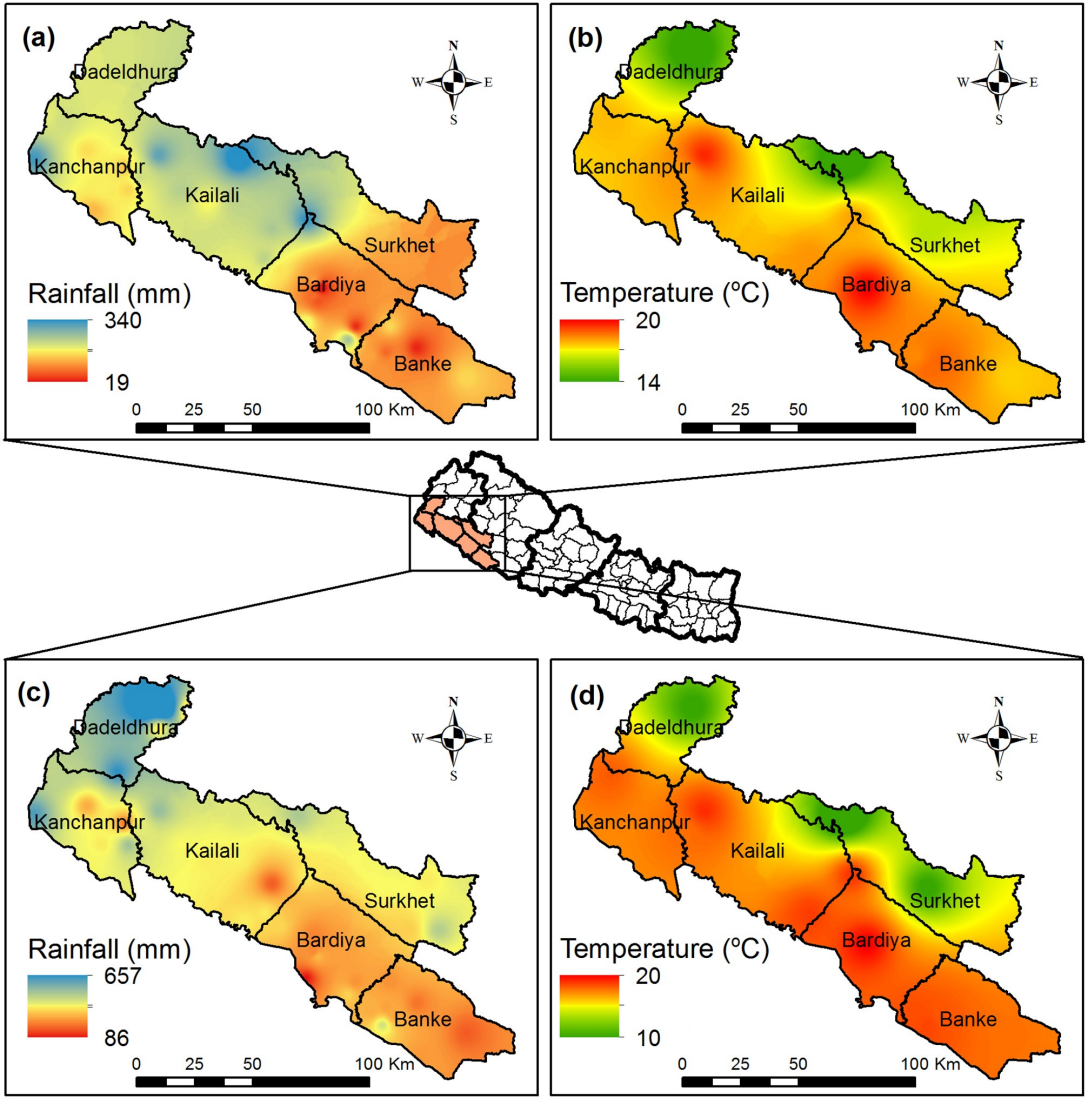
Within season and across year’s spatial variability in climatic parameters during lentil producing time (Oct–March) in study areas: a) rainfall in 2014, b) temperature in 2014, c) rainfall in 2015, and d) temperature in 2015.

The spatial distribution of rainfall and average temperature during lentil production season for surveyed years are presented in Fig 1. There was a high within and across seasonal variability in rainfall among sampled districts. Winter rainfall variability in 2014 ranged from 19 mm to 340 mm, while the variability widened to 86 mm to 657 mm in 2015. There was less variability in average temperature during surveyed years (Fig 1). Long-term trend analysis for seasonal rainfall shows a positive slope indicating winters are becoming wetter (S1 Fig). Similar results were reported in earlier studies conducted in western Nepal [40,75].

### Regression results

#### Model specification tests

In order to test potential multicollinearity among the explanatory variables, variance inflation factors (VIFs) were calculated. Myers [76] suggested that VIFs values for each of the explanatory variables should not cross the limit of 10, in order to be free from multicollinearity. The mean VIF for the stochastic frontier model was 2.10 and ranged from 1.06 to 5.92, indicating no signs of multicollinearity. Additionally, the VIF value ranges from 1.09 to 7.18, with a mean value of 2.12 in the case of the Tobit model, indicating no signal detection for multicollinearity. Furthermore, the Cobb-Douglas type production function in agriculture is expected to follow a constant returns to scale (CRS), i.e., doubling all of the inputs increases the output by the same proportion [77]. However, since environment and/or climate related variables (rainfall and temperature) are not under the farms direct control and doubling these inputs may result excessive waterlogging and/or extreme drought in crop leading to crop senescence, we excluded these variables while conducting test against constant returns to scale. This means that the sum of partial production elasticities should be one and the test on respective regression coefficient failed to reject the null hypothesis that their sum is one, indicating the existence of constant returns to scale in the production function.

#### Parameter estimates in the stochastic production function for lentil farming in western Nepal

The maximum likelihood estimates for the parameters in the stochastic frontier model as defined in Eq (2) are presented in Table 3. The slope coefficient defines the output elasticity of inputs and the estimated signs of parameters are as expected. The result from maximum likelihood estimates for the parameters in the stochastic frontier model and Tobit model indicated that lentil production in the wet years was significantly and negatively affected by higher rainfall and waterlogging (Table 3). These results indicate that a 1% increase in seasonal rainfall decreases lentil yield by 0.40% in the stochastic frontier model and the impact magnitude is even higher (1.57%) in the Tobit model. There is a significant interaction effect of rainfall with land type on grain yield, where a 1% increase in rainfall in lowland areas decreases lentil yield by 0.22% in the stochastic frontier model and by 0.36% in the Tobit model (Table 3). The stochastic frontier model shows that farms that suffered waterlogging had a 16.7% lower yield and the impact magnitude in the Tobit model is much higher at 43.6%. A more modest temperature effect was observed with a 1% increase in the average temperature decreasing lentil yield by 1.25% in the stochastic frontier model and by 3.4% in the Tobit model.

**Table 3:** Maximum likelihood estimates of the parameters in the stochastic production frontier for lentil production in western Nepal.

Variables	Stochastic frontier model		Tobit model	
	Coefficient	Std. error	Coefficient	Std. error
*Production frontier*				
Lentil land (ha)	0.798***	0.075	0.397***	0.121
Labor (hours)	0.069**	0.036	0.153**	0.063
Non-labor capital (NRs.)	0.019**	0.009	0.060***	0.016
Seed (kg)	0.147***	0.057	0.310***	0.102
Nitrogen (kg)	0.040***	0.015	-0.111***	0.025
Phosphorus (kg)	-0.018	0.014	-0.021	0.023
Potash (kg)	-0.038	0.021	-0.033	0.031
Rainfall (mm)	-0.400***	0.116	-1.571***	0.204
Waterlogging (1 = yes)	-0.183**	0.089	-0.572***	0.147
Temperature (° C)	-1.257***	0.473	-3.382***	0.857
Variety (1 = improved)	-0.246*	0.132	-0.491**	0.225
Relay cropping (1 = yes)	-0.495***	0.150	-1.180***	0.249
Mixed cropped (1 = yes)	-0.067	0.090	0.198	0.145
Tillage method (1 = tractors)	0.110	0.095	0.333**	0.152
Diseases (1 = yes)	-0.379***	0.093	0.182	0.156
Sandy soil^†^ (1 = sandy)	0.085	0.102	0.516***	0.175
Clay soil (1 = clay)	0.459***	0.100	0.632***	0.150
Lowland (1 = lowland)	0.324***	0.102	-0.013	0.168
Rainfall × lowland	-0.221***	0.024	-0.360***	0.041
Constant	12.01***	1.828	20.25***	3.286
$\sigma_{v}^{2}$	0.536***	0.052		
Wald/LR ch^2^ [19]	701.54		357.03	
Log likelihood	-916.85		-1414.07	
*Technical inefficiency*				
Cultivated land (ha)	0.351***	0.101		
Household size (no)	-0.005	0.018		
Gender of household head (1 = male)	0.017	0.169		
Farming experience (years)	-0.020***	0.006		
Labor availability (1 = easily available)	-0.242*	0.141		
Wage rate (NRs)	0.174***	0.043		
Lentil specialized farms (1 = yes)	0.859***	0.167		
Mean technical efficiency (TE) score	0.408			
*TE in upland (N = 261)*	*0*.431*			
*TE in lowland (N = 404)*	*0*.*395*			
No. of observations	665		793	

*** indicates significant at 1% level,

** indicates significant at 5% level and * indicates significant at 10% level.

^†^The base category is the silt soil. TE stands for technical efficiency. Exchange rate: 1 US $ = NRs 98.6 and NRs. 102.7, respectively, for year 2014 and 2015 [86].

The stochastic frontier model showed that the occurrence of disease in lentil fields significantly reduces grain yield. Farmers who reported the presence of disease had a 31.5% lower lentil yield than the farmers who did not report disease problems. However, the coefficient of disease was not significant in the Tobit model (Table 3). Among the farmers who do not lose their crop, 78% reported incidence of disease and harvested 206 kg ha^-1^ lower yields. In general, farmers could not identify the specific diseases that affect their fields; based on observations from the pathologists from the Nepal Agricultural Research Council (NARC), *Stemphyllium* leaf blight is the most common and damaging disease of lentil in western Nepal.

As expected, our findings are consistent with economic theory that shows that the partial production elasticities for land, labor, and capital inputs are positively associated with output, and these variables are statistically significant (*P*< 0.05) in both of the models. However, the magnitude of the elasticities for land, labor, and capital varies across two models. While the magnitude of elasticities for land is larger in the stochastic frontier model (0.798) than in the Tobit model (0.397), the elasticities for labor and non-labor capital are smaller in the stochastic frontier model than in the Tobit model. Seed rate and soil type had a significant positive effect (*P <* 0.01) on lentil productivity in both models. Yield reduction was significantly higher in the field where improved seed varieties were grown. Similarly, seeding method had significant effect on grain yield, where grain yield was significantly lower for farmers who practiced relay seeding rather than other seeding methods.

Further, the model coefficient for nitrogen is positively associated with yield in the stochastic frontier model, while this coefficient sign is reversed in the Tobit model, likely because proportionally more farmers who applied nitrogen also experienced crop failure. Since this association is unlikely to be causal since applied fertilizer rates were low in all surveyed fields, we consider estimates from production function are more reliable than those from the Tobit model.

#### Parameters estimates for technical efficiency

The parameters for the technical efficiency model as specified in Eq (2) are presented in Table 3. In order to correctly interpret the efficiency determinants, it should be noted that the dependent variable is the one-sided error term reflecting technical inefficiency. Therefore, positive signs of the coefficient indicate technical efficiency-reducing factors and negative signs reflect technical efficiency-enhancing factors. Our results showed that the farm holding size, years of farming experience, farm labor availability, labor wage rate, and production specialization affect the technical efficiency of lentil production (Table 3). The results showed that more experience in lentil farming and timely availability of farm labor increases technical efficiency.

Similarly, technical efficiency in lentil production decreased with increases in the on-farm wage rate. Our results are plausible because when the on-farm labor wage rate increases, propensity to use labor decreases due to capital constraints, which ultimately affects crop management practices and technical efficiency. In the same vein, household labor availability is positively associated with technical efficiency. We also found strong evidence that technical efficiency increases with decreasing farm size (cultivated land). Surprisingly, lentil-specialized farms are negatively associated with technical efficiency.

The histogram of the technical efficiency score estimated from the stochastic frontier model showed that on average lentil producing farms are 41% efficient (S2 Fig), indicating significant scope for improvement with the caveat that factors such as drainage class are best viewed as intrinsic field characteristics that can only partially be modified by management. In wet winters, technical efficiency is higher in upland ecologies (43%) than in the lowland ecologies (39%).

### Results from on-farm trials

Multi-locational trials were conducted across all districts in the region of interest over a five-year period (i.e. crop harvests in 2012, 2013, 2014, 2015, and 2016) to explore how crop establishment practices (zero tillage), local modification of drainage (bed planting), choice of lentil variety, and integrated better bet agronomic management (for example: improved variety, alternative seeding method, timely weed management, fertilizer management etc.) affect yield and yield stability across years. None of interventions or their interactions were statistically significantly, hence we combined data across all treatments to explore location and year effects.

Lentil yields varied across the years and production ecologies. Across western Nepal, wet winters were experienced in lentil season of 2012–13 and 2014–15 with poor yields attained in lowland ecologies (i.e. grain yield < 300 kg ha^-1^) (S2 Table). However, yields in upland sites (Dadeldhura and Surkhet) exceeded 1.0 t ha^-1^. In the drier winter years of 2015–16 and 2016–17, lentil yielded more than 1.2 t ha^-1^ in Terai ecologies, while there was no or very poor yields in the upland sites in the mid-hills (S3 Fig). All of the results from on-farm experiments indicated that in wet winters, lowland areas are risky for lentil production and upland areas have high yields, a dynamic that is reversed in dry winters.

### Simulating *Stemphylium* blight disease risk in lentil

**Fig 2:**
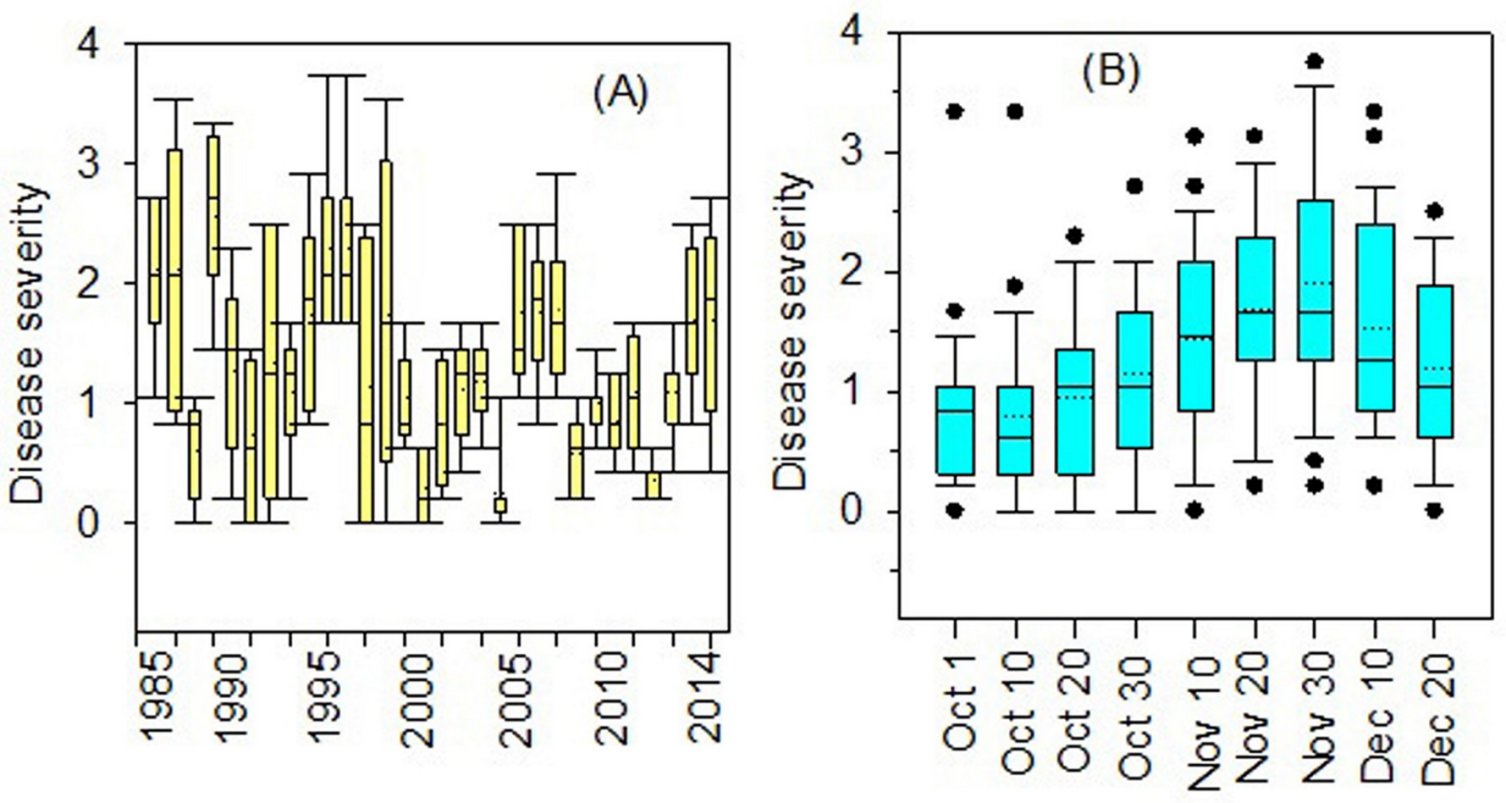
*Stemphylium* blight simulations with the stempedia model with 30 years of historical weather data: A) severity across years, B) severity across years as a function of planting time.

Simulation results from the Stempedia model driven by historical climatic data (1985–2014) confirms that *Stemphylium* disease risk varies across years but that 73% of years are likely to have significant *Stemphylium* blight disease outbreaks in fields where inoculum is present (Fig 2A). These results are consistent with the stochastic frontier model that has shown a negative effect of diseases with lentil production (Table 3) and *Stemphylium* blight could be a major reason. Moreover, it is important to note that local differences in drainage class and soil type influence the likelihood of disease, but these factors are not simulated in the Stempedia model and the result are best interpreted as general trends as governed by weather conditions. Results also suggest that disease severity increases with delayed crop establishment, i.e., seeding after the 1^st^ week of November (Fig 2B). This suggests that the risks posed by *Stemphylium* blight can be greatly reduced through earlier planting in October.

## Discussion

Although development efforts in Nepal have highlighted the importance of pulse crop intensification to food, nutritional, and income security, our results confirm that lentil remains a risky enterprise with survey data from two seasons highlighting the prevalence of crop failures (16%), modest yields (353 kg ha^-1^), and low levels of profitability (US$ 33 ha^-1^), particularly in wet winters. Nevertheless, site factors such as drainage class influence responses with upland sites performing well in wet winters and lowland sites performing well in dry winter (S3 Fig). The dominant production area for lentil in Nepal is in the Terai plain where poorly drained conditions are common.

Lentil is particularly sensitive to the direct effects of waterlogging and moisture deficit during flowering and pod-filling stage [48,78]. Although available soil moisture and rainfall are important for lentil production in South Asia, waterlogging in lentil also damages the root systems thereby limiting crop capacity for water uptake when conditions become drier [79]. Perhaps more importantly, significant negative effects of high rainfall can increase disease severity, particularly for fungal diseases [80]. In our study, 78% of farmers reported significant disease incidence in their fields in the comparatively wet winters of 2014 and 2015 (Table 1) and disease incidence had a large negative impact on lentil production (Table 3). While, some wilt-related diseases are prolific when there is drought [81], other blight and fungal diseases are more prominent when there is high moisture in the soil and the broader environment [82,83]. Due to increasing winter precipitation levels in Nepal, the fungal disease *Stemphylium* blight has become a serious threat [49,50].

High and stable yields of lentil are only likely to be achieved in Nepal when the threats posed by diseases mediated by winter rainfall are effectively and economically managed. Although fungicides provide a plausible response strategy, they remain out of reach for most farmers due to cost and limited availability in the market. Through on-farm trials conducted across many sites in western Nepal, our study evaluated the potential role of alternative crop establishment (zero tillage), drainage modification (bed planting), and varietal choice among cultivars currently available in Nepal as possible entry points for increasing yield and yield stability. Unfortunately, statistically significant gains were not associated with any of these interventions. That said, work outside the region does suggest that there is significant genetic variation in lentil resistance to waterlogging tolerance [48] and *Stemphylium* blight that can be leveraged by bringing new lines to Nepal for testing and registration [84].

Perhaps most promising as a near-term intervention is the possibility of adjusting planting dates to reduce the risks posed by *Stemphylium*. Results from the Stempedia model simulations suggests that planting lentil within October can greatly minimize *Stemphylium* blight disease severity (Fig 2). Nevertheless, the majority of lentil in Nepal is grown in rice-based cropping system [35,36] and most of the rice varieties grown are medium to long-duration. To benefit lentil in rice-based systems, efforts are required to encourage earlier rice planting (i.e. permitting earlier rice harvest) and to facilitate transitions to shorter-duration rice varieties where there is an opportunity to do so while enhancing the performance of the cropping system as a whole.

Even without progress in addressing climate-based risks, our survey results suggest that lentil producing farms are only 41% efficient, with wide variability (0.2% to 85%) (S2 Fig). This indicates significant scope to increase lentil productivity and technical efficiency in Nepal through better practices, although the opportunity for efficiency gains are not uniform across production ecology gradients (e.g. drainage class). We observed a significant positive effects of farming experience on technical efficiency (Table 3), suggesting knowledge bottlenecks are constraining production. Hence, more extension efforts through public and private sector channels is likely warranted. On the other hand, limited labor availability and increasing on-farm wage rates have negative effects on the technical efficiency of lentil production and this could be the reason for negative association of lentil specialized farms with technical efficiency (Table 3). Moreover, in Nepalese context, labor out-migration has created an acute labor shortage that has affected the timely crop establishment. In this context, availability of scale-appropriate farm mechanization can be a viable option to cope with the problem of labor shortages that increases the labor wages and affect the farm productivity and technical efficiency. However, smallholder farmers may not be able to purchase farm mechanization due to capital constraint. They could, however, hire the mechanization services if service providers rent-out the services [85]. Hence, supporting the emergence of mechanized service provision through small and medium-sized entrepreneurs is a scalable means to address these downward forces on technical efficiency [85].

## Conclusion

Since the majority of lentil in Nepal is produced in the Terai plain of western Nepal, research and development efforts should first be focused on the unique challenges and opportunities present in this region. Based on the combined insights emerging from our survey, field trial, and simulation results, gains in yield, yield stability, and technical efficiency can be made by: 1) ensuring timely lentil planting to mitigate climate-mediated disease risk, 2) evaluating new lentil lines that may provide enhanced resistance to diseases and waterlogging, and 3) encouraging the emergence of mechanization through service provision. By addressing these foundational constraints, more farmers will likely be poised to adopt other good management practices. Finally, as the skill of seasonal weather forecast improves, provision of climate services information may provide a useful guide to farmers with respect to matching management intensity and investment to likely yield outcomes.

## Supporting information

S1 Fig Historic winter rainfall trends from selected meteorological stations in the Nepal Terai. (TIF)

S2 Fig Distribution of technical efficiency score for overall farms. (TIF)

S3 Fig Lentil yield (kg ha^-1^) from a series of on-farm evaluation across different ecologies and years in western region districts of Nepal from 2012/13-2016/17. (N inside the figure indicate the total number of samples included).
(TIF)

S1 Table Details on agronomic practices, experiment year, district and number of locations included in each district for on-farm experiments. (DOCX)

S2 Table Districts wise lentil yield and seasonal precipitation during lentil growing time (October-March) in study areas. (DOCX)

S1 Data (RAR)
