# Supplementary figures and images for: Climate and landscape mediate patterns of low lentil productivity in Nepal
Source: PLoS One. 2020 Apr 16;15(4):e0231377. doi: 10.1371/journal.pone.0231377 (PMC7162466; doi:10.1371/journal.pone.0231377)

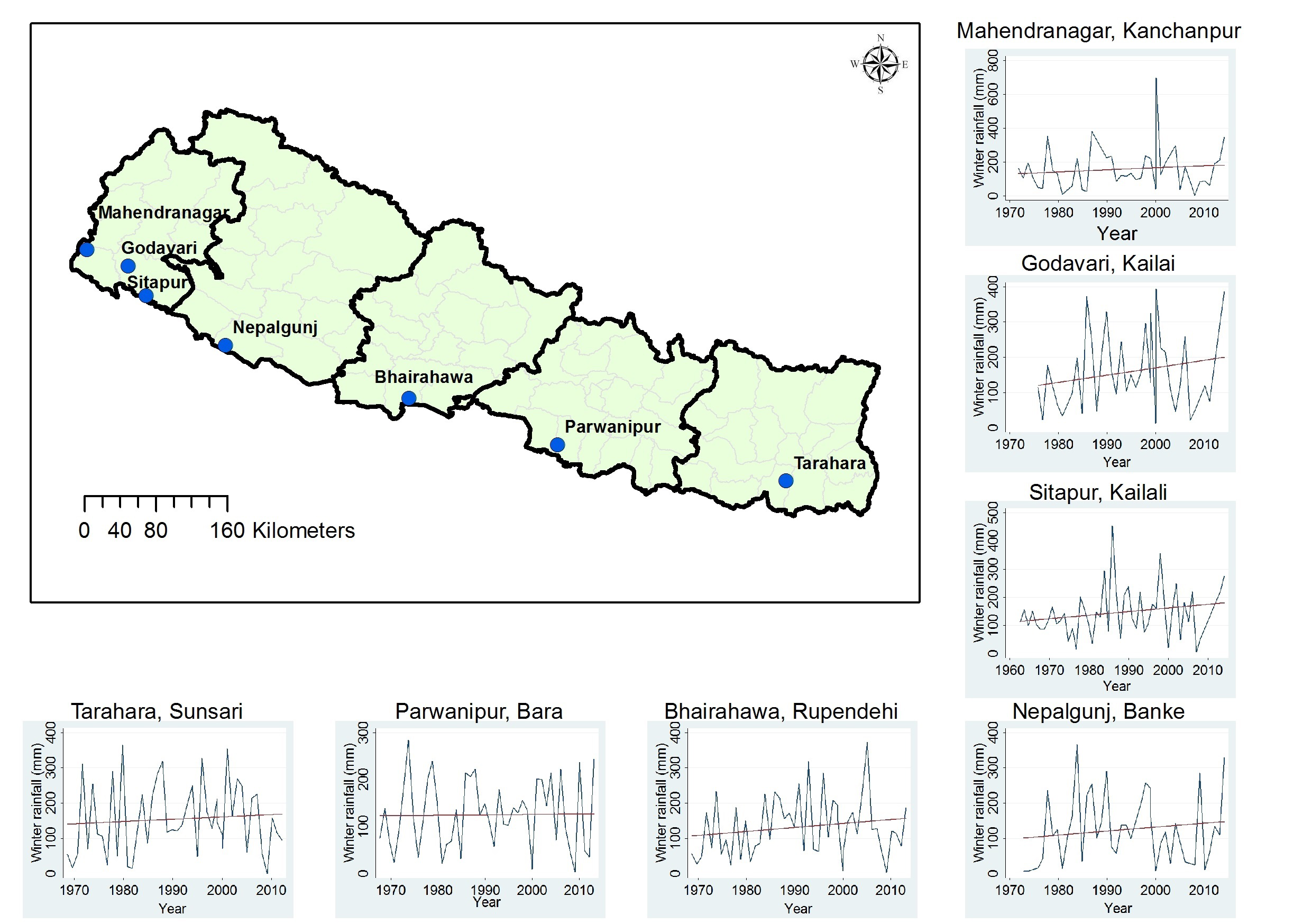

Supplement: S1 Fig — (TIF) [file pone.0231377.s001.tif]

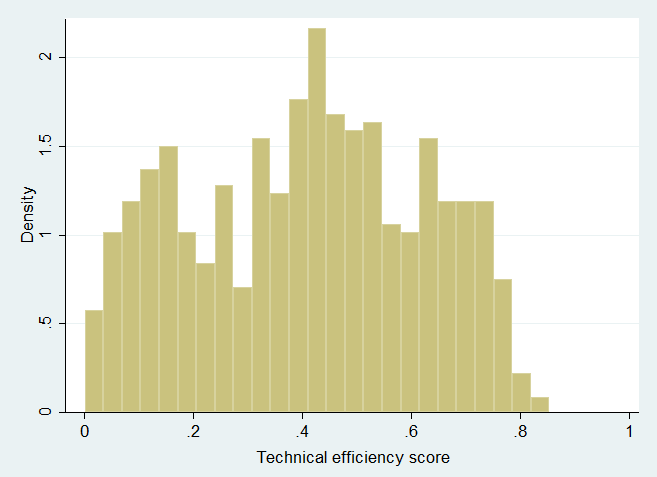

Supplement: S2 Fig — (TIF) [file pone.0231377.s002.tif]

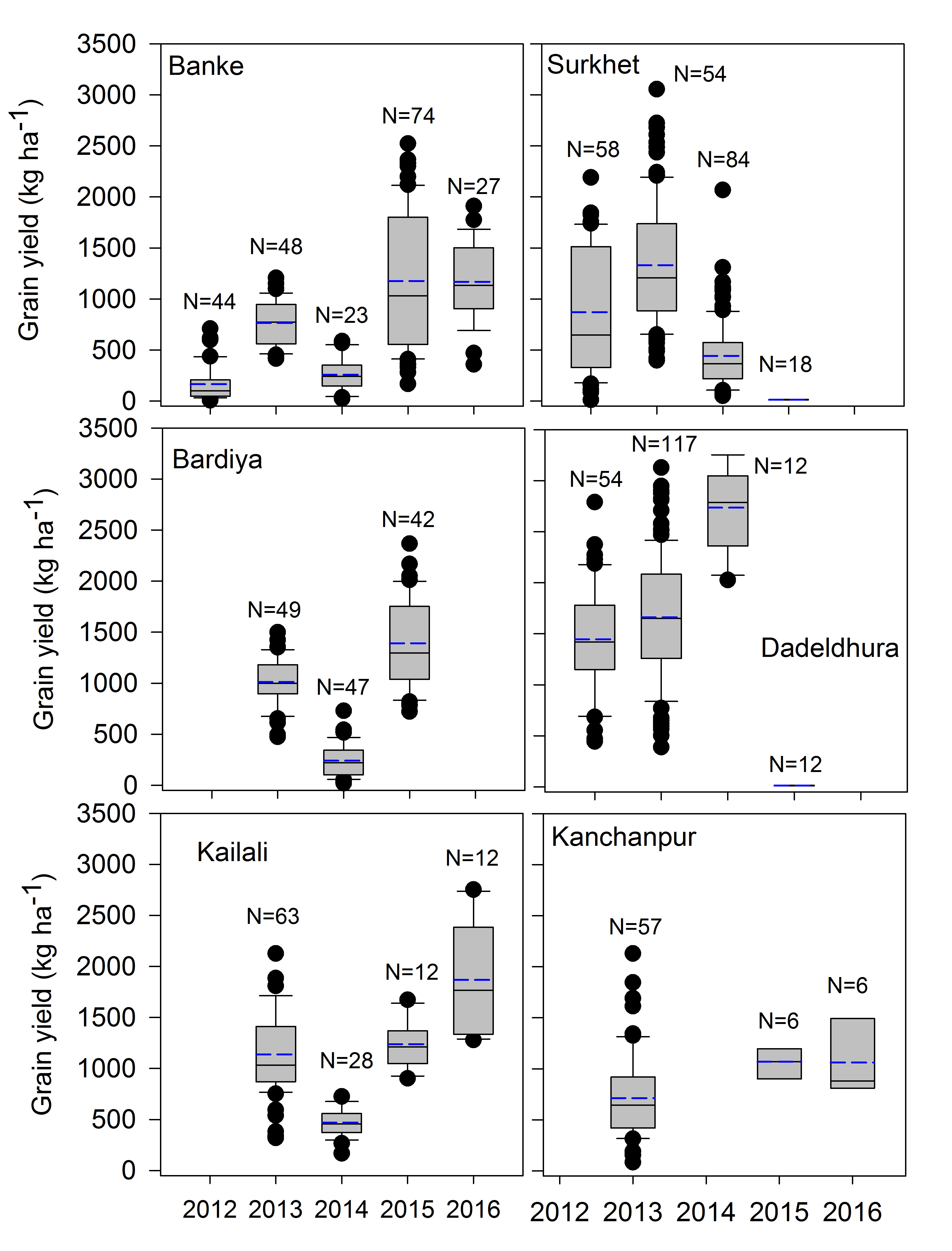

Supplement: S3 Fig — (N inside the figure indicate the total number of samples included). (TIF) [file pone.0231377.s003.tif]
