# Supplementary material for: Climate and landscape mediate patterns of low lentil productivity in Nepal
Source: PLoS One. 2020 Apr 16;15(4):e0231377. doi: 10.1371/journal.pone.0231377 (PMC7162466; doi:10.1371/journal.pone.0231377)
Supplement: S1 Table — (DOCX) [file pone.0231377.s004.docx]

**S1 Table. Details on agronomic practices, experiment year, district and number of locations included in each district for on-farm experiments.** Note: Numbers in the parenthesis indicates the number of locations in the respective year and districts.

| Practices | 2012/13 | 2013/14 | 2014/15 | 2015/16 | 2016/17 |
| --- | --- | --- | --- | --- | --- |
| Varietal evaluations | Banke (3), Surkhet (6) | Dadeldhura (3),  Surkhet (6) | Banke (3), Bardiya (3),  Kailali (3), Surkhet (10), Dadeldhura (3) | Dadeldhura (3), Bardiya (3),  Kailali (3), Kanchanpur (3), Banke (4) |  |
| Planting methods |  |  | Banke (3), Bardiya (6),  Kailali (4) | Banke (10),  Bardiya (6),  Kailali (4) Kanchanpur (3) | Banke (4), Kailali (3), Kanchanpur (3) |
| Integrated management | Banke (9), Surkhet (17) | Banke (8),  Bardiya (7),  Kailali (9), Kanchanpur (9), Dedeldhura (26), Surkhet (12) | Kailali (9), Surkhet (18) | Surkhet (7) |  |
