# Supplementary material for: Climate and landscape mediate patterns of low lentil productivity in Nepal
Source: PLoS One. 2020 Apr 16;15(4):e0231377. doi: 10.1371/journal.pone.0231377 (PMC7162466; doi:10.1371/journal.pone.0231377)
Supplement: S2 Table — (DOCX) [file pone.0231377.s005.docx]

**S2 Table. Districts wise lentil yield and seasonal precipitation during lentil growing time (October-March) in study areas.** Note: Only average values are displayed in the table for lentil yield and seasonal precipitation, for brevity.

| Year | Terai districts | | | | | | | | Hilly districts | | | |
| --- | --- | --- | --- | --- | --- | --- | --- | --- | --- | --- | --- | --- |
|  | Banke | | Bardiya | | Kailali | | Kanchanpur | | Surkhet | | Dadeldhura | |
|  | Yield (kg/ha) | Rainfall (mm) | Yield (kg/ha) | Rainfall (mm) | Yield (kg/ha) | Rainfall (mm) | Yield (kg/ha) | Rainfall (mm) | Yield (kg/ha) | Rainfall (mm) | Yield (kg/ha) | Rainfall (mm) |
| 2012 | 165 | 134 | – | – | – | – | – | – | 870 | 157 | 1445 | 210 |
| 2013 | 766 | 110 | 1012 | 63 | 1137 | 182 | 713 | 190 | 1332 | 286 | 1661 | 547 |
| 2014 | 258 | 329 | 241 | 194 | 470 | 289 | – | – | 442 |  | 2736 |  |
| 2015 | 1176 | 0 | 1392 | 0 | 1238 | 0 | 1069 | 0 | 7 | 0 | 5 | 0 |
| 2016 | 1168 | 26.5 | – | – | 1868 | 31.4 | 1061 | 33.2 | – | – | – | – |
